# Clinico-microbiological profile of urinary tract infection in south India

**DOI:** 10.4103/0971-4065.75226

**Published:** 2011

**Authors:** M. Eshwarappa, R. Dosegowda, I. Vrithmani Aprameya, M. W. Khan, P. Shiva Kumar, P. Kempegowda

**Affiliations:** Department of Nephro-Urology, M. S. Ramaiah Hospital, MSRIT Post, New BEL Road, Bangalore, Karnataka, India

**Keywords:** Community-acquired urinary tract infection, extended spectrum beta lactamase, antibiotic resistance

## Abstract

The knowledge of etiology and antibiotic resistance pattern of the organisms causing urinary tract infection is essential. This study was taken up to determine the presentation and risk factors associated with community-acquired urinary tract infection (CA-UTI). The distribution of bacterial strains isolated from these patients and their resistance pattern were also studied. This multidisciplinary prospective observational study was conducted in M. S. Ramaiah Hospital, Bangalore, between January and December, 2008. Patients who had CA-UTI confirmed by positive urine culture reports were included in the study. Statistical analysis was done using the SPSS version 16. Symptomatology and others risk factors for CA-UTI were studied in these patients and the causative organisms and their resistance patterns were recorded. Of the total 510 patients included, 57% belonged to the elderly age group (50–79 years). Fever and dysuria were the most common clinical presentation, but were not specific in predicting CA-UTI. Escherichia coli (66.9%) was the most common organism causing CA-UTIs with extended spectrum beta lactamase (ESBL) resistance seen in nearly two-thirds of these cases (42.2%). The organisms recorded least resistance against carbapenems (3.9%). A high resistance rate was seen for fluoroquinolones (74.1%). In conclusion, a high rate of ESBL-positive organisms and their resistance to commonly used antibiotics brings a concern for future options in treating these conditions.

## Introduction

Urinary tract infection (UTI) is one of the most common infectious diseases seen in the community.[1] Empirical antibiotic therapy is usually applied here and for this, knowledge of the common uropathogens and their susceptibility to commonly used antibiotics is needed. Treatment becomes even more challenging in the presence of risk factors such as higher age, comorbidity, and immunosupression. Many times, physicians resort to prescribing broad-spectrum antibiotics over specific antibiotics in the view of resistance of the causative organism to the antibiotic. Poor patient compliance and incomplete course of antibiotic therapy have resulted in the evolution of resistance to many of these antibiotics. Various studies done worldwide have shown changing patterns in the etiology of UTIs.[2,3] However, studies on UTI and the pattern of antibiotic resistance in India are few.[4,5] The present trends of the uropathogens and their susceptibility to various antibiotics are essential to formulate guidelines for the empirical treatment of UTIs while awaiting the culture sensitivity.

The aim of the present study was to record the common clinical presentation and risk factors for UTI. The distribution of bacterial strains isolated from complicated and uncomplicated UTIs occurring in the community and their resistance pattern against commonly used antibiotics at our setting were also studied.

## Patients and Methods

The study was done in M. S. Ramaiah Memorial Hospital, Bangalore, from January to December, 2008. The study included all the patients who were admitted or visited the out-patient department in the hospital with symptoms of UTI during the study period and had UTI confirmed by positive urine culture reports. Only one sample from each subject was considered. Subjects with clinical symptoms of UTI but samples not grown on any organism were excluded from final analysis. Patients who underwent treatment with another antimicrobial within 48 h or within 24 h if only a single dose and in the presence of an appropriate positive culture and ileal loops or vesicoureteral reflux were also excluded from the study. Data were collected using a questionnaire regarding demographic and clinical data. The subjects were classified as having complicated UTI based on the criteria defined by Rubenstein and Schaeffer [Table 1].[6]

**Table 1:** Identification of patients with complicated urinary tract infections[5]

Men
Children
Nosocomial infection
Women
Known lesion on prior diagnosis
Functional or structural urinary tract anomaly
Obstruction (e.g., stone, ureteropelvic junction obstruction)
Pregnancy
Diabetes
Spinal cord injury
Neurological disorders (e.g., multiple sclerosis) that affects bladder function
Indwelling catheter
Comorbidities that predispose to papillary necrosis (e.g., sickle cell disease, severe diabetes, analgesic abuse, Pseudomonas species infection)
Infection with an unusual organism (e.g., tuberculosis)
Suspected lesion based on history
Unresolved urinary tract infections – failed response to antimicrobial therapy
Bacterial persistence (recurrent urinary tract infections with the same organism)
Infection with urea-splitting organisms
Recurrent febrile urinary tract infections in childhood
Suspected lesion based on symptoms
Febrile urinary tract infections (especially >3 days)
Renal colic
Gross hematuria

### Isolation and identification of uropathogens

A clean-catch midstream specimen or suprapubic aspirate, in subjects who were unable to give the former, was collected in a sterile wide-mouth leak-proof container to hold about 50 ml specimen. Using a calibrated loop method of a loop diameter of 4 mm, 10 *μ*l of the uncentrifuged specimen was transferred onto the agar plate and streak using the modified Mayo’s technique without flaming the loop for isolation and incubated at 35–37°C for 24 h. A specimen was considered positive for UTI if a single organism was cultured at a concentration of >105 colony-forming units/ml. The Gram-positive and Gram-negative organisms were culture isolates which were further identified by using various biochemical reactions up to genus/species levels wherever applicable.

### Antibiotic sensitivity testing

In the presence of any potential growth, antibiotic sensitivity testing was done by the modified Kirby-Bauer disc diffusion method according to the Clinical and Laboratory Standards Institute (CLSI) guidelines.[7] The antibiotics tested were imepenem, meropenem, ciprofloxacin, ofloxacin, norfloxacin, amikacin, gentamicin, nitrofurantoin, and cotrimoxazole (Pathoteq Labs, India).

### Extended spectrum beta lactamase detection

The screening for extended spectrum beta lactamase (ESBL) was done using cefpodoxime (≤17 mm), ceftazidime (≤22 mm), aztreonam (≤27 mm), cefotaxime (≤27 mm), and ceftriaxone (≤25 mm). If the organisms showed a zone of inhibition lower than the minimum for any antibiotic disc, ESBL positivity was suspected. The phenotypic confirmation was done by testing the strain against ceftazidime (Ca) and ceftazidime/clavulanic acid. A >5-mm diameter of the zone of inhibition for ceftazidime/clavulanic acid in comparison to ceftazidime was considered indicative of ESBL production. *Escherichia coli* ATCC 25922 was used as an ESBL-negative and *Klebsiella pneumoniae* 700603 was used as an ESBL-positive reference strain.[7]

### Management of UTI

All hemodynamically stable patients with UTI were started on oral fluoroquinolones and cephalosporins. Hemodynamically unstable patients were started on third-generation cephalosporins parenterally. If the symptoms did not subside over next 72 h and the culture showed ESBL-positive organisms, then these patients were started on parenteral carbapenem therapy. All patients received antibiotic therapy for 7 days. If fever persisted after 7 days, antibiotic therapy was continued for 48 h after fever subsided.

### Statistical analysis

The analysis was done using the statistical software package SPSS version 16. Age, gender, organisms causing UTI, their antibiotic sensitivity and resistance, symptomatology of these patients and risk factors for UTI were included as variables in the model.

## Results

Overall, 5564 subjects suspected to have UTI were screened. Of these, 510 patients showed growth on the urine culture and were included in the study [Table 2]. The mean age was 52.84±22.25 years. Most of the cases were recorded in the elderly age group (50–79 years, 57.4%). Pediatric cases comprised 9.8% of the total cases. The mean age of complicated UTI patients was 55.47±21.51 years (95% CI 53.50–57.45 years) and was 29.69±13.76 years (95% CI 25.87–33.52 years) for uncomplicated UTI. As males were categorized as complicated cases, all 52 uncomplicated cases were females. In complicated UTI, the female:male ratio was 1:1.63.

**Table 2:** Age- and gender-wise distribution of complicated and uncomplicated urinary tract infection

Age group	Complicated UTI				Uncomplicated UTI		Total	
	Male	Percentage	Female	Percentage	Female	Percentage	Total	Percentage
0-9	22	7.7	3	1.7	5	9.6	30	5.9
10-19	8	2.8	8	4.7	4	7.7	20	3.9
20-29	17	5.9	10	5.8	20	38.5	47	9.2
30-39	18	6.3	8	4.7	7	13.5	33	6.5
40-49	24	8.4	10	5.8	13	25.0	47	9.2
50-59	57	19.9	39	22.7	3	5.8	99	19.4
60-69	64	22.4	39	22.7	–	–	103	20.2
70-79	53	18.5	38	22.1	–	–	91	17.8
80-89	18	6.3	14	8.1	–	–	32	6.3
90-99	5	1.7	3	1.7	–	–	8	1.6
Total	286	100.0	172	100.0	52	100.0	510	100.0

Most of the cases were recorded in the elderly age group (5079 years, 57.4%). Pediatric cases comprised 9.8% of the total cases

**Figure 1:**
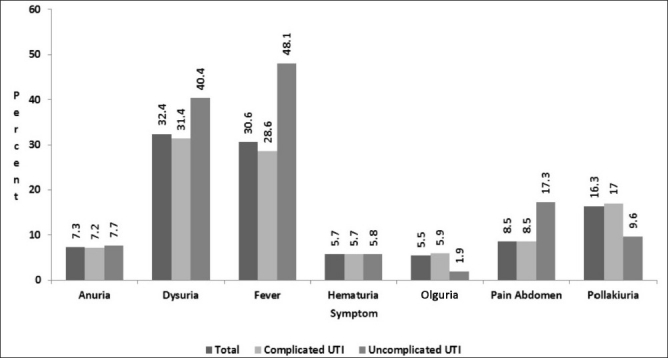
Various symptomatologies seen in patients with UTI during the initial presentation. Fever and dysuria were the most common presenting symptoms in the present study

Fever and dysuria were the most common clinical presentation of the patients in UTI, overall as well as individually in complicated and uncomplicated UTI [Figure 1]. Diabetes (42.6%) was the most common factor associated with complicated UTI in our study. While the recent history of urogenital instrumentation (TURP, cystoscopy, stenting) other than catheterization was present in 16.2% of the study subjects, catheterization alone posed a significant risk factor seen in 11.4% of them [Table 3].

**Table 3:** Frequency of risk factors in subjects with urinary tract infection

Risk factor	Frequency	Percentage
Catheterization	52	11.4
Congenital anomalies	21	4.6
Diabetes mellitus	195	42.6
Immunosuppression	3	0.7
Post-transplant status	9	2
Recent history of urogenital instrumentation	75	16.4
Recurrent urinary tract infection	40	8.7
Renal stones	9	2

Diabetes mellitus was the most common risk factor associated with UTI in the present study

ESBL-positive *E. coli* (42.2%), ESBL-negative *E. coli* (24.7%), ESBL-positive *K. pneumoniae* (9.6%), ESBL-negative *K. pneumoniae* (5.9%), and *Pseudomonas aeruginosa* (10.2%) were the most prevalent microorganisms in UTI patients [Table 4]. The frequency and distribution of the UTI pathogens were similar in complicated and uncomplicated UTI. ESBL-positive *E. coli* (43.9%) was the most common causative organism in complicated cases, while ESBL-negative *E. coli* was found in 50% of uncomplicated cases.

**Table 4:** Frequency and distribution pattern of pathogens and percentage of ESBL production

Organism	Complicated	Percentage	Uncomplicated	Percentage	Total	Percentage
*Citrobacter freundii*	13	2.8	0	0	13	2.5
*Enterobacter spp.*	10	2.2	2	3.8	12	2.4
*Enterococcus faecalis*	7	1.5	1	1.9	8	1.6
ESBL-positive *E. coli*	20	43.9	14	26.9	215	42.2
ESBL-negative *E. coli*	100	21.8	26	50	126	24.7
ESBL-positive *Klebsiella*	44	9.6	5	9.6	49	9.6
ESBL-negative *Klebsiella*	29	6.3	1	1.9	30	5.9
Morganelle	1	0.2	0	0	1	0.2
*Proteus vulgaris*	1	0.2	0	0	1	0.2
*Providencia alkalifaciens*	1	0.2	2	3.8	3	0.6
Pseudomonas	51	11.1	1	1.9	52	10.2
Total	458	100	52	100	510	100

Gram-negative organisms were the most common organism causing UTI in the study

The antimicrobial potency and spectrum for nine selected antimicrobial agents of different classes against the UTI pathogens recorded in the study are summarized in Table 5. Carbapenems had the least resistance (3.9%), followed by amikacin (28.0%), and nitrofurantoin (28.6%). A high rate of resistance was recorded against quinolones (74.1%). The antibiotic resistance pattern in complicated UTI was similar to that in overall infection with carbapenems having the least resistance (4.1%), followed by amikacin (29.0%) and nitrofurantoin (31.2%). However, organisms in uncomplicated UTI showed lesser resistance toward nitrofurantoin (5.8%).The mortality associated with CA-UTI in the present study was 8.4%. The mortality in patients having ESBL-positive UTI was 9.8%.

**Table 5:** Resistance pattern of the uropathogens to various antibiotics

Organism	Complicated	Percentage	Uncomplicated	Percentage	Total	Percentage
Amikacin	133	29.0	10	19.2	143	28.0
Ciprofloxacin	352	76.9	26	50.0	378	74.1
Cotrimoxazole	156	34.1	1	28.8	171	33.5
Gentamicin	233	50.9	18	34.6	251	49.2
Imepenem	19	4.1	1	1.9	20	3.9
Meropenem	19	4.1	1	1.9	20	3.9
Nitrofurantoin	143	31.2	3	5.8	146	28.6
Norfloxacin	352	76.9	26	50.0	378	74.1
Ofloxacin	352	76.9	26	50.0	378	74.1

Nearly three-fourths of all the isolated samples were resistant to quinolones

## Discussion

Although UTI ranks among the most common infection in developing countries, in the present study, only 510 of the 5564 suspected cases (9.17%) were proved by culture. This indicates that urine culture is essential for a definitive diagnosis of UTI. The low culture positivity rate in the present study could be partly explained by nonspecific symptoms such as fever and pain abdomen. We found that the presence of combinations of these symptoms has a better chance to be UTI rather than the lone symptoms.

Increased frequency was the most common symptom among acute uncomplicated UTI in a study done by Little et al.[8] Sepahi et al.[9] found that fever, pain, irritability, dysuria, and hematuria were the main clinical presentation of UTI in the presence of urolithiasis in children. Similar clinical symptomatology was seen in the present study. But the predictability of UTI by these symptoms is to be questioned. Individually, none of the symptoms were potent enough to pick up most of the UTI. When two or more symptoms were taken in combination, their predictability was still very low (fever and dyuria 11.4%, fever and pollakiuria 2.2%). The presence of a risk factor (complicated UTI) marginally improved the predictability of these symptoms to diagnose UTI (diabetes and fever 7.1%, diabetes and dysuria 12.2%, diabetes, fever, and dysuria 1.6%). These findings indicate that clinical presentation plays a very minor role, if any, in diagnosing UTI, reconfirming the fact that urine culture is essential to diagnose UTI.

Diabetes mellitus has a number of long-term effects on the genitourinary system. Diabetic nephropathy is one of the many factors that make these patients more susceptible to UTI than nondiabetics. Reduced immunity in diabetes also contributes to the increased risk for acquiring UTI. A range of infrequent presentations of complicated UTI such as emphysematous pyelonephritis and emphysematous pyelitis are commonly seen in patients with diabetes. Because of their predisposity to these uncommon presentations, there are recommendations for including plain radiograph and ultrasonography of abdomen while investigating UTI to look for upper urinary tract involvement.[10]

For either short- or long-term catheters, the infection rate is about 5% per day.[11] Infection spreads by biofilm formation on both internal (intraluminal route) and external (periurethral route) catheter surface. Despite precautions, the majority of patients catheterized for >2 weeks eventually develop bacteriuria.[12] Asymptomatic bacteriuria is the most common presentation of catheter-associated UTI. Current recommendations are not to treat asymptomatic catheter-associated UTI as it leads to the emergence of drug-resistant organisms.[11] To prevent infection, intermittent catheterization by either a nurse or by the patient is advised.[12]

The uropathogen profile in our study is similar to other studies.[4,5,13,14] Contradicting findings have been reported regarding the uropathogens’ profile and their antibiotic sensitivity patterns in the presence of diabetes. While Stapleton[15] found that the organisms causing UTI in diabetic patients are significantly different than those in nondiabetics, Bonadio *et al*.[16] reported that diabetes made no difference to the uropathogen profile or the antibiotic sensitivity pattern. Similar results were seen in the present study.

Kader *et al*. reported 8.9% ESBL-positive cases in a hospital-based study in Saudi Arabia.[17] Bean *et al*. reported a community-based ESBL prevalence to be 5.7% in London.[18] In the present study, 52.2% of the isolates were ESBL-positive uropathogens. Previous studies in India have reported an ESBL positivity rate between 26.9% and 48.3%.[4,5,19,20] ESBL producers do not respond to the usually prescribed empirical therapy. Also, there is an increased risk of associated morbidity and mortality, and cost of therapy when these patients are put on the standard empirical therapy.[21] Presently, alternative antimicrobial therapy to treat ESBL-positive UTI on outpatient basis is limited. Carbapenems are the most effective in this situation[22] but need to be administered intra-venously or -amuscularly. The experimental use of fosfomycin in treating ESBL-positive UTI has also shown promising results in the recent past.[23,24] All this and the high rate of ESBL positivity in the present study warrant a change in the empirical therapy for UTI to prevent the complications.

The antibiotic susceptibility pattern in the present study is similar to other studies.[25–29] Quinolones were the least active drug against uropathogens in the present study. The resistance rate for ciprofloxacin has been increasing over decades and this is the highest resistance rate reported to date. Akram *et al*.[4] reported ciprofloxacin resistance rates ranging from 47% to 69% among the Gram-negative organisms in their study in India. Though the bacterial spectrum causing community-acquired UTI (CA-UTI) remained the same over time, the antibiotic susceptibility has changed. Prais *et al*.[30] studied bacterial susceptibility to oral antibiotics in CA-UTI in 1991 and 1999. They reported that the pathogens recovered in the two groups were similar but there was a generalized decrease in bacterial susceptibility to common antibiotics in the two groups. Although, quinolones were considered as one of the drugs of choice for the treatment of UTI, the increasing resistance rate necessitates a change in the empirical treatment against CA-UTI.

The uropathogens showed highest sensitivity to carbapenems. The next best alternatives were aminoglycosides. But again, nearly one-third and more than half of the uropathogens showed resistance against amikacin and gentamicin, respectively. Also, the carbapenem-resistant organisms, although only a few in the present study, raise a concern over the available options to treat complicated and drug-resistant cases. Until recently, carbapenems were almost uniformly active against resistant Gram-negative organisms, but some strains have now developed very effective ways to deal with the carbapenems. There are various mechanisms by which these organisms achieve such feat, by producing beta lactamases which destroy the antibiotics, by blocking the entry of these antibiotics, or by efflux pumps which actively pump out these antibiotics.[31] Furthermore, some of these mechanisms are not antibiotic or class specific, and can also be easily transferred from one organism to another. The situation is worsening everyday as no new antibiotics against these multidrug-resistant organisms are in advanced stages of clinical development.

With limited options and all the above-mentioned growing concerns, it would not be late where we will find ourselves in epidemics with multidrug-resistant organisms. We now have find alternative and economical options to fend off an otherwise catastrophe. Micek *et al*.[32] reported an improved outcome in sepsis when patients were put on empiric antibiotic therapy rather than the conventional monotherapy. A similar trial in treating UTI is yet to be evaluated. Kristensen and group[33] evaluated a Decision Support Group in a small Danish County in deciding the empirical treatment of bacteraemic urinary tract infection and found that a decision theoretic approach showed promise of improving empirical antibiotic treatment, and may be a measure to support an antibiotic policy. Such feat on a larger scale could help in establishing standardized empiric therapy. But care should be taken to include the prevalent organism and antibiotic susceptibility pattern of the region as it varies over larger geographic areas due to various reasons.

### Limitation

The phenotypic confirmation of ESBL-positive organisms was done using only ceftazidime/clavulanic acid and not cefotaxime/clavulanic acid as per the latest CLSI guidelines. As a result, there may be underreporting of the incidence of ESBL organisms in the present study.

## Conclusion

Clinical presentation plays a minor role in establishing diagnosis in UTI. Diabetes and urogenital instrumentation were the major risk factors for UTI. *E. coli* is still the most widely prevalent organism causing UTI in the community, only that the alarmingly high rate of resistant ESBL species should draw our attention. The resistance pattern, though not that different from the rest of the world, is ever increasing due to uncontrolled abuse of the available antibiotics. A strong decision has to be established regarding the antibiotic policies for UTI and stringent measures have to be taken to ensure the effectiveness of the same. Failing to do so, the time is not far where we would have to stand helplessly against these organisms.
